# Hypophosphatemic rickets is associated with disruption of mineral orientation at the nanoscale in the flat scapula bones of rachitic mice with development^☆^^☆☆^

**DOI:** 10.1016/j.bone.2012.04.021

**Published:** 2012-09

**Authors:** A. Karunaratne, G.R. Davis, J. Hiller, C.T. Esapa, N.J. Terrill, S.D.M. Brown, R.D. Cox, R.V. Thakker, H.S. Gupta

**Affiliations:** a Queen Mary University of London, School of Engineering and Material Sciences, Mile End Road, London, E1 4NS, UK; b Queen Mary University of London, Barts and the London School of Medicine and Dentistry, Institute of Dentistry, E1 2AD, UK; c Diamond Light Source Ltd., Diamond House, Harwell Science and Innovation Campus, Chilton, Didcot, Oxfordshire, OX11 0DE, UK; d Academic Endocrine Unit, Nuffield Department of Clinical Medicine, Oxford Centre for Diabetes, Endocrinology and Metabolism (OCDEM), University of Oxford, Churchill Hospital, Headington, Oxford, OX3 7JL, UK; e MRC Mammalian Genetics Unit and Mary Lyon Centre, MRC Harwell, Harwell Science and Innovation Campus, OX11 0RD, UK; f Department of Chemistry, University of Sheffield, Dainton Building, Brookhill, Sheffield, S3 7HF, UK

**Keywords:** Hpr, hypophosphatemic rickets, SAXS, small angle X-ray scattering, LB, lateral border, IF, infraspinous fossa, ENU, N-ethyl-N-nitrosourea, PBS, phosphate buffered saline, *χ*, predominant direction of orientation, *ρ*, degree of orientation, N-ethyl-n-nitrosourea, X-linked hypophosphatemic rickets, Scanning synchrotron small angle X-ray scattering, Mineralisation, Bone mineral properties

## Abstract

Metabolic bone disorders such as rickets are associated with altered *in vivo* muscular force distributions on the skeletal system. During development, these altered forces can potentially affect the spatial and temporal dynamics of mineralised tissue formation, but the exact mechanisms are not known. Here we have used a murine model of hypophosphatemic rickets (Hpr) to study the development of the mineralised nanostructure in the intramembranously ossifying scapulae (shoulder bone). Using position-resolved scanning small angle X-ray scattering (SAXS), we quantified the degree and direction of mineral nanocrystallite alignment over the width of the scapulae, from the load bearing lateral border (LB) regions to the intermediate infraspinous fossa (IF) tissue. These measurements revealed a significant (*p* < 0.05) increase in mineral nanocrystallite alignment in the LB when compared to the IF region, with increased tissue maturation in wild-type mice; this was absent in mice with rickets. The crystallites were more closely aligned to the macroscopic bone boundary in the LB when compared to the IF region in both wild type and Hpr mice, but the degree of alignment was reduced in Hpr mice. These findings are consistent with a correlation between the nanocrystallites within fibrils and *in vivo* muscular forces. Thus our results indicate a relevant mechanism for the observed increased macroscopic deformability in rickets, via a significant alteration in the mineral particle alignment, which is mediated by an altered spatial distribution of muscle forces.

## Highlights

► During murine scapulae development, mineral nanoplatelet alignment and content increase faster in the periphery compared to the intermediate region. ► In the rachitic condition, nanoscale mineral orientation and direction are altered together with altered muscle forces and reduced mineralisation. ► Nanostructural mineral parameters may mediate fracture and deformability in rickets via association between muscle forces and mineral alignment.

## 1 Introduction

Physiological forces generated by muscles and tendons play an important role in the formation and maturation of bone tissue, as illustrated by studies examining the link between forces and mineralised nanostructure on load-bearing long bones such as femur or ulna [1–4]. For example, investigations of a mouse model for hypophosphatasia have revealed that defective mineralisation is associated with significant changes in the nanostructure of long bones, from a gradual decrease in orientation along the axis to a more random distribution [4]. Long bones, which are formed by endochondral ossification, are primarily uniaxially loaded in the mid-diaphysis (although significant torsional forces are present at the extremities, for example at the femoral head). However, the impact of such forces on the formation of flat bones such as scapulae, ilia or calvariae is not known. Flat bones develop by intramembranous ossification, in which mesenchymal cells aggregate, differentiate into osteoblasts and begin to produce bone extracellular matrix; the spatial distribution of muscle forces across these flat bones is often complex and multiaxial [5]. Furthermore, the impact of disease-induced disruptions of the mineralisation process on the spatial-temporal development of bone nanostructure [6–8] in the multi-axial force regime of flat bones, and their biomechanical consequences, remain to be determined. We therefore undertook studies to elucidate these structural and mechanical processes using small-angle X-ray scattering (SAXS) analysis on murine scapula bone.

SAXS provides information on the arrangement of nanostructural mineral crystallites [9], as well as the collagen fibril orientation. In contrast, techniques such as micro-computed tomography (micro-CT) analysis, quantitative back scattered electron microscopy and dual-energy X-ray absorptiometry do not provide information on nanostructural components of the bone matrix, as they are spatially limited in resolution to approximately 1 μm. Moreover, scanning SAXS, where a micron-scale X-ray beam provides a 2D raster of SAXS images, has been applied to map micro- and nanoscale heterogeneities in bone tissue [2], and to characterise mineral crystal changes with development [9], disease-induced disruptions of nanostructure [4] and structure at the bone-implant interface [10]. These studies showed that local mechanical forces are critical in controlling mineral particle orientation in long bones, with elongated mineral particles in the mid shaft of murine ulnae oriented along the long axis a few weeks after birth, an effect absent in the (load-free) embryonic mouse femora [2]. Evidence of greater mineral alignment close to implanted tantalum devices and gradients in mineral crystallite thickness have also been shown, and these have been attributed to local mechanical forces that were induced by the implant material [1]. These studies support the idea that alterations at different hierarchical levels in bone are induced by *in vivo* mechanical stimulation.

These nano- and microstructural bone mineralisation patterns will be significantly altered in metabolic bone diseases, which would in turn alter the transduction of the *in vivo* mechanical load that would result in changes to the force distribution locally. These changes in force distributions would be expected to subsequently alter the tissue development, via mechanotransduction to the osteoblasts, osteoclasts and osteocytes [11,12], and thus lead to alterations in bone formation. To study such interactions between alteration of bone matrix quality in metabolic bone disorders with *in vivo* force distributions and tissue growth, we selected rickets, which is associated with decreased mineralisation due to deficiency of vitamin D, phosphorus or calcium [13]. This impaired mineralisation has been shown to alter the bone material quality and the functional biomechanics of the tissue at micro- [14] and nanoscale levels [15]. In this study, we have used an N-ethyl-N-nitrosourea (ENU) induced mouse mutant for X-linked hypophosphatemic rickets (Hpr), arising from a Trp314Arg missense mutation in the *Phex* (phosphate-regulating gene with homologies to endopeptidases on the X-chromosome) gene [15], and focused our studies on the scapula for the following reasons.

The scapula is a large triangular flat bone which has five thick bony ridges (glenoid, scapula spine, medial and lateral border (LB) and caracoid process) and two hard flat bony structures, denoted as infraspinous fossa (IF) and supraspinous fossa [5]. The scapula is subject to a number of muscle, ligament and joint reaction forces during movement, and the location, magnitude and direction of these forces differ extensively between tissue regions within the same scapula. Indeed, the force variation at different muscle insertion points can be very large, with a spatially variable stress distribution ranging from 0.05 MPa at IF versus 60 MPa at LB estimated using finite element modelling at the macroscale [5]. We therefore utilised the scapula from Hpr mice as a model system to investigate muscle force-mediated mineral particle orientation and its alteration due to defective bone mineralisation, using synchrotron scanning X-ray nanoimaging methods. Advances in synchrotron X-ray sources generate X-ray beams of micrometre size (1–10 micrometres), allowing scanning SAXS experiments to map spatial variations in the nanostructure with a high resolution [16]. This technology enables quantitative investigation of the nanocrystallite organisation in the tissue, with micron-scale resolution.

## 2 Materials and methods

### 2.1 Animals

An ENU induced mouse model for X-linked hypophosphatemic rickets (Hpr) arising from a missense Trp314Arg *Phex* mutation was used [15]. Wild-type and Hpr male mice aged 1, 4, 7 and 10 weeks were studied. Mice were kept in accordance with UK Home Office welfare guidelines and project licence regulations.

### 2.2 Sample preparation for Scanning Small Angle X-Ray Scattering

**Fig. 1:**
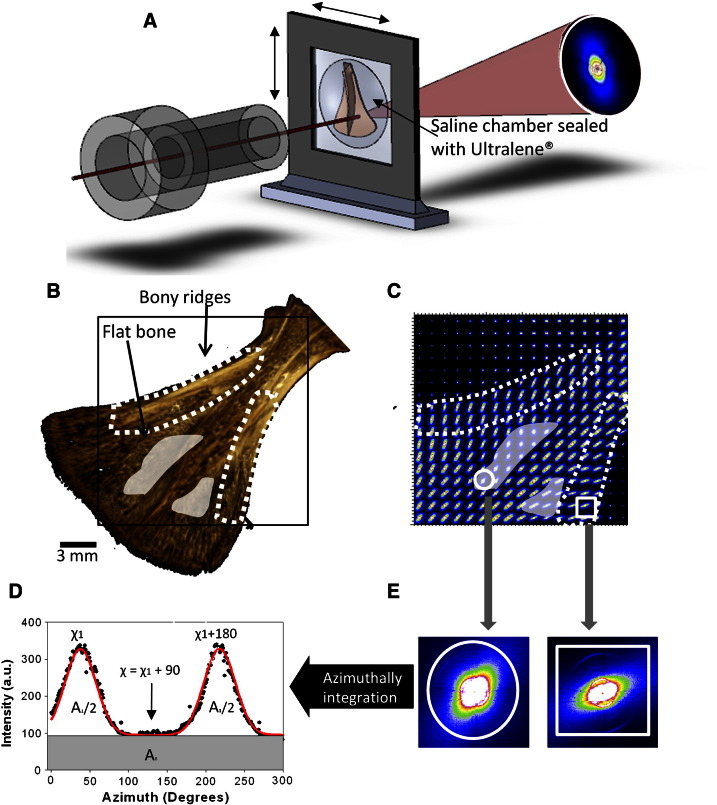
Scanning synchrotron SAXS on mouse scapula and the systematic data reduction procedure: (A) Schematic of scapula mounted in a saline sample chamber sealed by Ultralene® foils. Black solid line shows the incident X-ray beam (~ 200 μm radius). A representative SAXS image is shown on the right. Scanning composite maps are built up by translating the chamber horizontally and vertically. SAXS images were collected every 200 μm on a 3.4 × 3.4 mm^2^ area of scapula. (B) Three dimensional rendered micro-CT image of scapula from a 4 week-old male wild-type (WT) mouse. Bony ridges and flat bony areas denoted by dotted lines and shades light grey patches respectively. (C) A composite map of SAXS images obtained by scanning across the scapula bone shown in (B), showing the variability between regions of high (white dash lines) and low (white transparent areas) muscle force. (D–E) shows schematically the data reduction in going from a 2D SAXS image (inset) to a 1D profile by azimuthal integration from which the angle (*χ*) and degree of orientation (*ρ*) can be obtained. The direction of the primary X-ray beam is perpendicular to the plane of the drawing.

Dissected scapulae from 1, 4, 7 and 10 week old mice were skinned, cleaned of muscle tissue, wrapped in gauze, soaked in phosphate buffered saline (PBS), and stored at − 20 °C until the scanning SAXS experiment was conducted (approximately 1 week). Just before the experiment, each scapula was mounted in a saline sample chamber with Ultralene® (SPEX SamplePrep, Metuchen, NJ, USA) foil windows, as shown in Fig. 1(A). For the scanning SAXS measurements of specimens, 3.4 mm^2^ (Fig. 1(B)) areas were selected. Due to the limited beam-time available for synchrotron measurements, only one scapula was chosen per age-point and disease condition for the scanning SAXS experiments.

### 2.3 Synchrotron scanning small angle scattering of scapulae

**Fig. 2:**
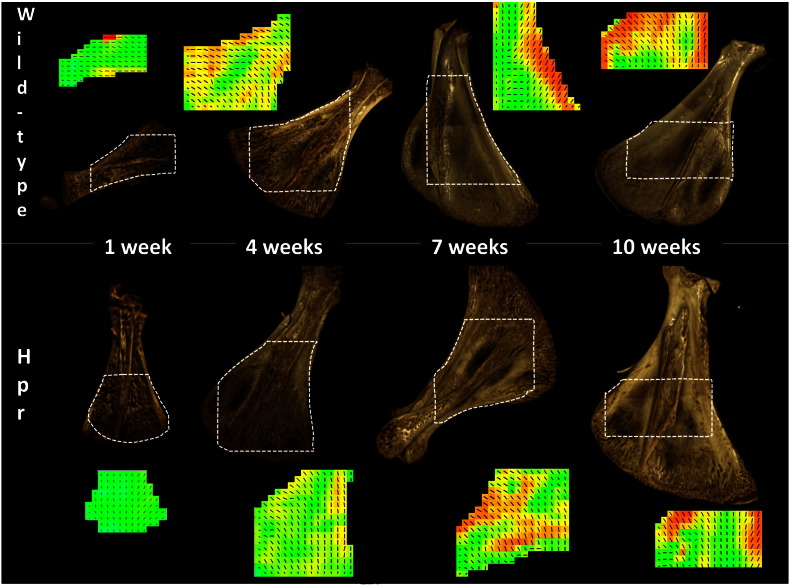
Abnormal development in Hpr mice scapula: Plots (top row—left to right) show the 3D rendered images from micro-CT and their 2D composite maps combining predominant orientation (direction of the lines) and degree (black = high degree of alignment, white = low degree of alignment and lengths of the lines) of mineral nanocrystallite orientation in normal mice. Plots (bottom row left to right) show the same for Hpr mice. In both cases, surface porosity decreases with age, but less pronounced in Hpr. The overall degree of orientation, as indicated by the greyscale and lengths of the lines, increases with age. However while the degree of orientation in wild-type mice increases at the bony ridges at the edge of the scapulae, in Hpr mice the increment is not to the same extent.

The mouse scapulae (1 sample at each time point) were contained in sealed sample chambers. These were mounted on a 2-axis motorised linear stage on beamline I22 at the Diamond Light Source (Harwell, Oxfordshire, United Kingdom). A schematic of the setup is shown in Fig. 1(A). A synchrotron X-ray beam (wavelength *λ* = 1.24 Å, beam cross section 200 μm × 200 μm) was used to measure the SAXS patterns. SAXS patterns were collected on a 2D multiwire RAPID2D detector system [17]. The distance between the sample and the detector was 7.57 m, which was verified with a calibration standard. Each SAXS data frame had a pixel resolution of 512 × 512 pixels and a pixel size of 383.4 × 383.4 μm^2^. Exposure time for a single SAXS image was 10 s. SAXS patterns of scapulae were collected in a raster map Fig. 1(C) of 3.4 × 3.4 mm^2^ with a step size of 200 μm in both vertical and horizontal directions. The scanning composite SAXS map (Fig. 2) of the mouse scapula, which illustrates the distribution of the measurement positions, was obtained by translating the chamber horizontally and vertically. Two dimensional SAXS images were analysed as described previously by Rinnerthaler et al. [18]. We determined two numerical parameters from each SAXS pattern in this study: the predominant direction of orientation (*χ*) and the degree of orientation (*ρ*) of mineral particles; both these parameters reflect the collagen fibril orientation and direction, and thus give an indication of the local nanostructural characteristics of the bone tissue.

**Fig. 3:**
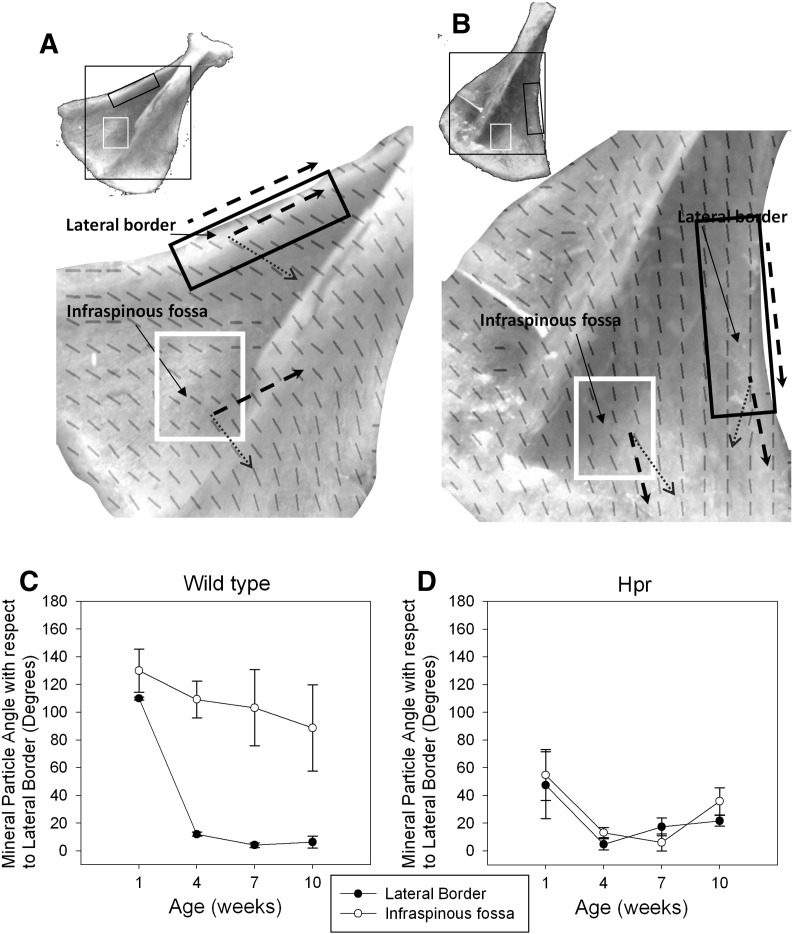
Analysis of direction of predominant orientation of mineral crystals with respect to the LB of individual scapulae. (A) Wild type 4 week old mice scapulae and (B) Hpr mice scapula with their composite map of mineral particle orientation showing the two anatomical regions of the LB (black box) and the IF (white box) that are subjected to different levels of muscle force, which were selected (insets) for relative measurement of the angle of the mineral particles. Direction (dash arrow) of LB was used as a reference line for angle measurements. (C) In wild type mice, mineral particles at the LB of the scapula become more aligned to the LB compared to the IF with age. The standard deviation increases with development in IF vs. LB, indicating an increase in randomness of mineral particle orientation in the regions experiencing lower muscle forces (IF). The increase in angle of the mineral particles in the IF may be due to lateral angular growth of the scapulae edges. (D) By contrast, the pattern observed in wild type mice is completely absent in Hpr mice.

The predominant direction of orientation of mineral particles can be derived using the *χ* parameter, as the particles are aligned along the main axis of the collagen fibril [18]. Scattering intensity is plotted as a function of the azimuthal polar angle (*χ*) (Fig. 1(D)) for a SAXS pattern in Fig. 1(E). Two peaks are separated by 180° (dark grey curve is the fitted Gaussian curves with centres separated by 180°). *χ*_1_ and *χ*_1_ + 180 are maximum scattering intensities. The direction of the mineral crystal long axis is defined by the angle *χ* = *χ*_1_ + 90. We compared the *χ* parameter at the LB (bony ridge; Fig. 3(A) black box) and the IF (flat bone; Fig. 3(A) white box) in the same scapula, to illustrate the impact of different muscle forces on the direction of mineral particle orientation. To ensure comparability, the same anatomical regions were selected in scapulae for all ages, in both wild type and Hpr mice. In order to compare the angle of the mineral crystals between scapulae of different ages, the direction of the LB (Fig. 3(A–B) black dashed line) of each scapula was used as a reference line.

SAXS data from the same two distinct tissue regions in the same scapula (Fig. 3(A–B)) were used to quantify the degree of orientation (*ρ*) of the mineral crystals, as previously defined by other researchers [18]. The parameter *ρ* indicates the anisotropy of the SAXS pattern defined as *ρ = A*_1_/(*A*_1_ *+ A*_0_), where *A*_0_ and *A*_1_ are the total SAXS intensity caused by randomly oriented mineral particles and particles aligned parallel to a certain direction, respectively (Fig. 1(D)). If there is no overall orientation within the plane of the scapulae, then *ρ* = 0; if all crystals are perfectly aligned, then *ρ* = 1.

### 2.4 X-ray microtomography measurements

X-ray microtomography was used to obtain tomograms (3 samples at each time point and disease condition); these were used to calculate degree of mineralisation at the micro level in scapula bone. A high-definition MuCat scanner [19] was used, comprising an X-tek ((Tring, Hertfordshire, UK), now part of Nikon Metrology (Leuven, Belgium)) ultrafocus X-ray generator and Spectral Instruments (Tucson, Arizona, USA) 800 series CCD camera in a time-delay integration readout mode. Scapula samples were scanned using an accelerating voltage of 40 kV and voxel size of 15 × 15 × 15 μm^3^. Following a calibration procedure, the micro‐CT projection data were corrected to 25 keV monochromatic equivalence and then reconstructed using a cone-beam back-projection algorithm to form a 3D image. Volume-rendered images (Fig. 1(B)) were produced to analyse the surface structure of the scapula.

Tomograms were also used quantitatively to assess the degree of mineralisation in the LB and the IF with increasing developmental age. Grey levels in the tomograms represent the linear attenuation coefficient (*μ*) of the sample, which was related to the degree of mineralisation in bone by the following relationship:

$${\text{Mineral}}_{\text{conc}}=\frac{\mu -\mu_{\text{o}}}{\mu_{\text{p}}-\mu_{\text{o}}}\rho_{\text{s}}$$

In this equation, *μ*, *μ*_o_, and *μ*_p_ are the measured, pure organic and pure sample material linear attenuation coefficients, respectively, and *ρ*_s_ is the sample material density.

The tomograms were converted into a series of 15 μm thick 2D bitmap stacks using Tomview software (in-house software of GRD). The histogram of the mineral concentration, denoted as the degree of mineralisation, was normalised against the bone volume of the sample and calculated for the two regions of interest, the LB and IF, using ImageJ software (ImageJ, NIH, USA). The weighted average mineral concentrations were determined from the degree of mineralisation of the LB and IF, and plotted as a function of developmental age and genotype.

### 2.5 Statistical analysis

To compare SAXS parameters for different ages at the same anatomical region, ANOVA single factor tests were performed. For example, to compare the change of SAXS parameters at the lateral border region of the tissue with development (from 1 week to 10 weeks), a single factor ANOVA test was carried out. Student *t*-test was performed between two different ages (e.g. 1 and 4 weeks) at an anatomical region. Excel 2007 (Microsoft Office 2007) was used for the ANOVA and Student *t*-tests.

## 3 Results

The bony ridges (LB) and the flat regions (IF), with high and low muscle forces acting respectively, are indicated in Fig. 1(B). A representative composite map (Fig. 1(C)) revealed variability in 2D SAXS patterns obtained from the different regions of the scapula, specifically between the IF (shaded) and the LB (dotted) regions. SAXS patterns obtained from the LB (Fig. 1(B); dotted region) showed a more elongated ellipsoidal intensity distribution (Fig. 1(C) square inset), compared to the SAXS patterns (Fig. 1(C) circle inset) taken from the LS (white shaded area). This ellipsoidal intensity distribution is due to variations in the electron density on roughly the 1–100 nm length scale; in the case of bone this results from the presence of plate-like mineral crystallites [20]. Highly parallel plate-like mineral crystals generate an anisotropic SAXS pattern, where the long axis in reciprocal space is perpendicular to the long axis of the crystallite in real space. Conversely, randomly oriented mineral crystals result in an isotropic SAXS pattern. Our results show clear differences in the degree of anisotropy of the SAXS patterns between bony ridges and flat bone regions within the same scapula, indicating a variation in degree of alignment of the mineral crystallites between anatomical sites. In order to quantify the predominant orientation (crystal angle (*χ*)) and degree of orientation (*ρ*), 1D plots (Fig. 1(D)) were obtained by azimuthal integration of the individual 2D SAXS images (Fig. 1(E)).

The 3-dimensional rendered images (derived from micro-CT measurements) of wild type and Hpr mice (Fig. 2 top and bottom row, respectively) revealed that surface porosity decreases with age (1 weeks to 10 weeks in Fig. 2) in both wild-type and Hpr mice. From a qualitative standpoint, however, this reduction was less pronounced in Hpr mice. Spatially resolved nanostructural data were obtained by scanning SAXS (Fig. 2) on the selected areas represented by the dashed polygonal sectors, and used to calculate mineral crystallite degree and direction of orientation at each point. This revealed that mean degree of orientation increases with age, by the increase in the lengths of the lines and the grey-scale (colour online) intensity level of the maps. In the wild-type mice, the most marked increase in degree of orientation occurs at the bony ridges at the edges (LB) of the scapula. Within the Hpr mice scapulae, in contrast, the degree of orientation does not increase to the same extent in either bony (LB) or flat (IS) regions.

### 3.1 Spatial variation of average direction of mineral nanocrystallites

We plotted the mineral particle angle Fig. 3(C–D) with respect to the LB as a function of age for both wild-type (Fig. 3(C)) and Hpr mice (Fig. 3(D)) scapulae. The variation in angle of the mineral particles with respect to the LB decreases with increasing age in wild-type mice scapulae (Fig. 3(C)). A significant (*p <* 0.001) reduction in the angle from ~ 110° to ~ 10° (a 90% reduction) occurs at the LB between 1 week and 4 weeks in wild-type mice scapulae. This is subsequently stabilised from 4 weeks to 10 weeks. In contrast, at the IF, there is no such reduction from 1 week to 4 weeks, and an overall decrease of only ~ 30% between 1 and 10 weeks.

However, these developmental changes in mineral crystallite angle of orientation, with respect to the scapula border, are quite different in the Hpr mice scapulae (Fig. 3(D)). Specifically, the developmental change in mineral particle angle with development is similar for both LB and IF. Starting from a lower degree of misalignment (~ 60°) at 1 week (compared to ~ 110–130° for wild type mice), the decrease of angle in both anatomical regions is similar (~ 85%). A subsequent slight increase is not statistically significant (*p >* 0.05).

### 3.2 Degree of mineral nanocrystallite alignment

**Fig. 4:**
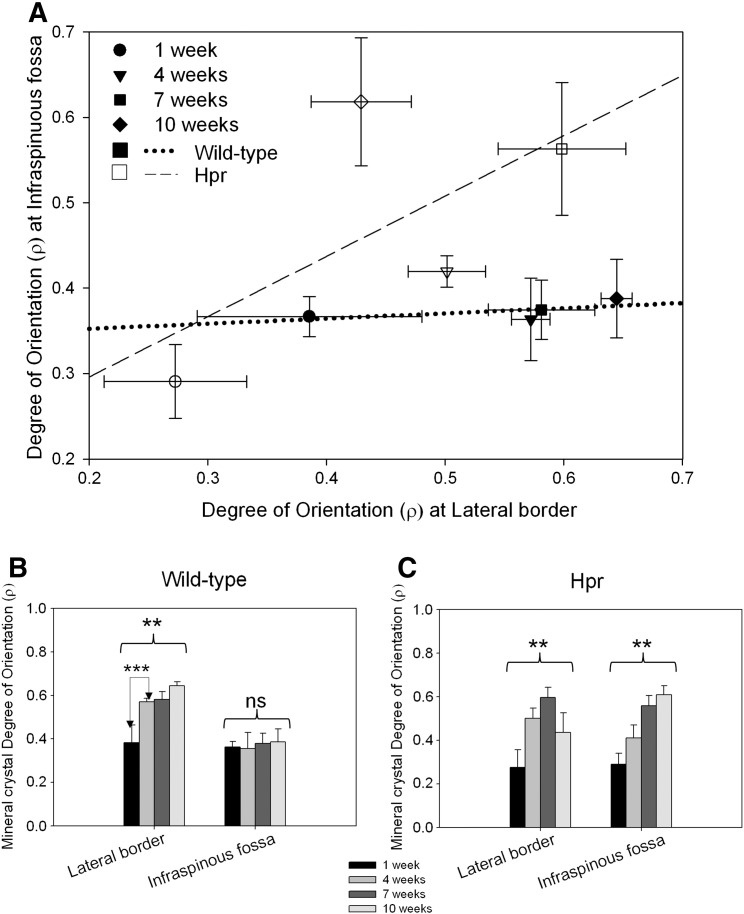
Development of degree of orientation, spatially resolved between the LB and IF*.* Degree of orientation in wild-type mice scapulae increases dramatically with age in LB (bony ridges) compared to IF (flat bone) shown by dotted line. However the most significant increase in degree of orientation is between 1 and 4 weeks of age. In contrast, in the Hpr mice, the overall degree of orientation increases with age, but the difference between LB and IF is completely absent. Likewise, the sharp increment from 1 week to 4 weeks (and subsequent stabilisation) in normal mice is completely absent in the Hpr mice. One-way ANOVA tests were carried out at both IF and LB to study variation of degree of orientation with age. Pair-wise brackets denote statistical significance (*p < 0.05, **p < 0.01, ***p < 0.001, ns: not significant).

Fig. 4 shows the values of *ρ* as a function of anatomical region and disease condition for all developmental ages. In the wild-type animals (Fig. 4A dotted line), the degree of orientation in the LB (bony ridge) increased significantly with age (*p* < 0.01). The most significant increment in the degree of orientation (*p* < 0.01) was observed between 1 week and 4 weeks in wild-type mice scapulae (Fig. 4B). After 4 weeks of age, the degree of orientation does not increase to the same extent. In contrast, at the IF, no statistically significant difference in degree of orientation with age was observed (*p* > 0.05). In Hpr mice (Fig. 4A dash line), in contrast, the degree of mineral crystallite orientation in both regions increases significantly (IF and LB: *p* < 0.01) (Fig. 4C). Intra-sample *t*-tests show that the significant increase is from 1 to 7 weeks for both regions (*p* < 0.01). Therefore, the difference between the LB and the IF is lost. These results showed that, in wild-type mice scapulae, the degree of orientation of the mineral crystals is greater at sites where higher muscle forces are exerted.

### 3.3 Degree of mineralisation

**Fig. 5:**
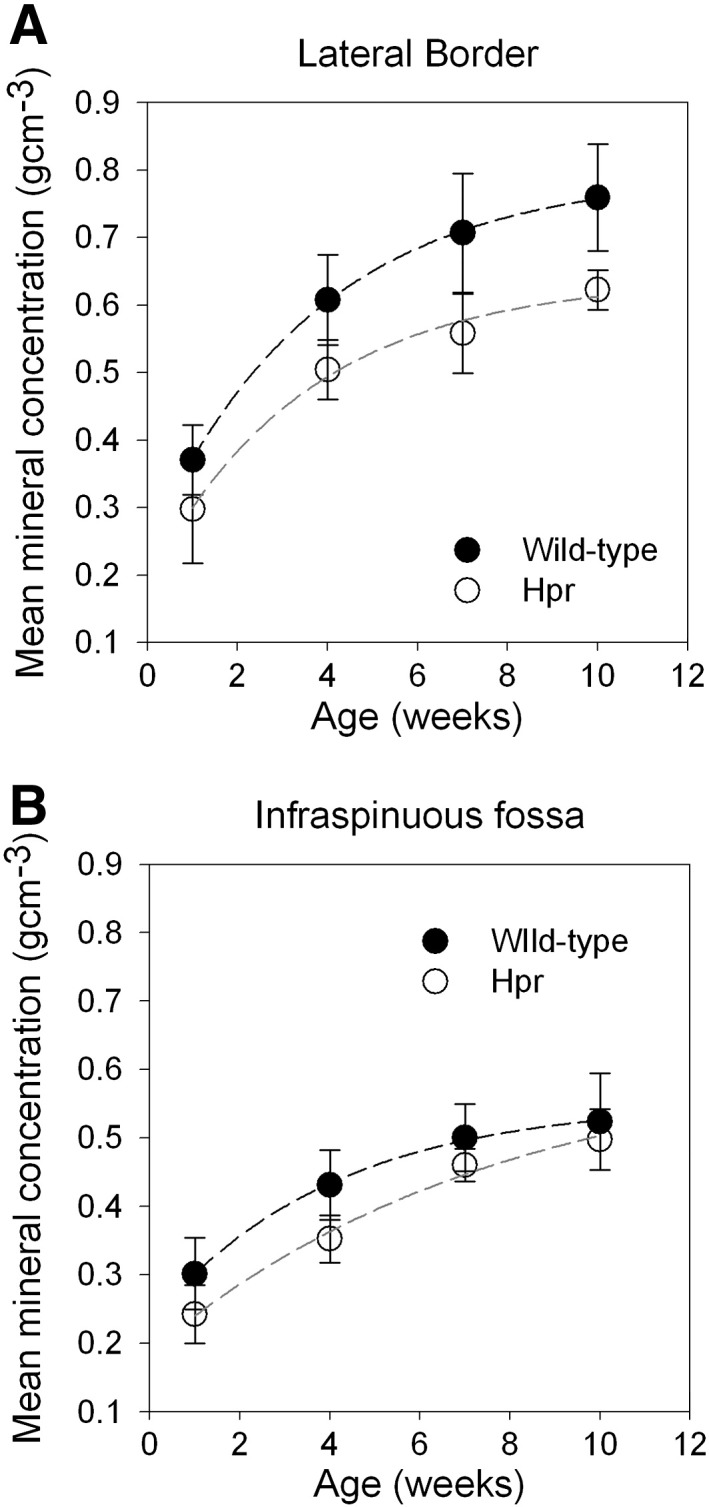
(A) Average mean mineral concentration of wild type mice scapulae plotted as a function of age from 1 week to 10 weeks for LB (filled symbols) and IF (open symbols). Lines are best fits (mean mineral concentration = *a* (1 − exp (− *b* × Age) + *c*) where *a*, *b* and *c* are constants, and are intended as guides to the eye only (*r*^2^ = 0.99)). (B) In the Hpr mice (*n* = 5), the increment in mineralisation with age is similar in both LB (filled symbols) and IF (open symbols). Lines are best fit curves (mean mineral concentration = *a* (1 − exp (− *b* × Age) + *c*) where *a*, *b* and *c* are constants, and are intended as guides to the eye only, (*r*^2^ = 0.99)). A significant difference in mineralisation rates between LB and IF was observed only in wild type mice (*p* < 0.05 and Hpr *p* > 0.05). Overall, mineral content at the IF is lower than the LB in both wild type and Hpr mice.

From the histograms of degree of mineralisation (measured using micro-CT), the mean mineral concentration was plotted as a function of age for LB (Fig. 5(A)) and IF (Fig. 5(B)), for both wild type and Hpr mice. The mean mineral concentration in wild type and Hpr mice was similar at 1 week for both the LB and the IF (Fig. 5(A)). The rate of increase in mineralisation with age was greater in wild type mice compared to Hpr mice (Fig. 5(B)) in the LB. However, in the flat infraspinous region, the rates of increase were similar for wild type and Hpr mice. The mean mineral concentration was lower at the IF compared to the LB in wild type mice at every age, and the difference became more significant (*p* < 0.05) with age. These variations across the scapula in wild-type mice show that increase in mineral content with age was greater at sites where higher muscle forces are exerted.

## 4 Discussion

From the foregoing, it is evident that our results demonstrate an association between muscular forces acting on the bone, and bone-matrix nanostructure with development in intramembranously ossified bones, and that a significant disruption of this correlation occurs under the conditions of hypomineralisation [21] and reduced muscular forces [22] observed in murine models of rickets. With scanning synchrotron SAXS [18], we were able to map microscale variations in bone nanostructure at different stages of tissue maturity. Scanning SAXS has previously been used to show reduction in mineral particle quality in diseases such as osteogenesis imperfecta [3], hypophosphatasia [4], and excess of fluoride dosage [23]. For example, in a murine model of osteogenesis imperfecta, small crystals with a greater variability in alignment were observed in cortical long bone, which may contribute to the brittleness in this condition. Moreover, the spatial pattern of mineral particle alignment, which is found to be highest in the femoral cortical midshaft and decreases toward the metaphyses and systematically increases with age in wild-type mice, is lost in TNALP-deficient mice, which is a model for hypophosphatasia; these changes could be due to a disruption of a highly ordered metaphyseal bone matrix due to ongoing remodelling in the cortical midshaft [4]. Scanning SAXS has also been used to analyse the nanostructure of human osteoporotic bone treated with sodium fluoride, and the mineral density, particle size and orientation of the resulting fluorotic bone were all found to exhibit differences compared to healthy bone [23].

However, the temporal and spatial variation of the mineralised nanostructure in bones such as the scapula, which are formed by intramembranous ossification, and where complex muscle-mediated forces act on the bone [5], along with disruption of these mineralisation mechanisms in metabolic bone diseases, has not been previously investigated. A better understanding of these mineralisation dynamics is clinically and biomechanically relevant because altered muscular forces have been shown to increase fragility [24]; moreover, skeletal deformability has been shown to increase in bone disorders mediated by weaker muscle forces, such as muscular dystrophy [25] and hypophosphatemic rickets [26]. Scanning SAXS has provided a unique perspective on understanding these mineralisation dynamics. The degree and direction of mineral particle predominant orientation observed here (Fig. 3(C–D)) give a measure of the organisation within the mineralised bone matrix at the nanoscale. Mineral crystallites closest to the regions of greater and unidirectionally oriented muscle forces, such as the LB, are more aligned to the LB in both wild type and Hpr mice, compared to crystallites in the flat IF region, which are subjected to lower and more multiaxial force. We further propose that in wild-type intramembranously ossifying bone, rapid alignment in the mineral phase occurs early in murine development, associated with the rapid growth of skeletal muscles and their elevated movements during the early postnatal period (1–4 weeks) [27]. Such an alignment would account for the observed large reduction in angle between mineral particle predominant orientation and a reference line at the LB in wild type mice between 1 and 4 weeks of age, as well as the subsequent stabilisation from 4 weeks to 10 weeks. However, in *Hpr* bones, this change in mineral particle alignment is much lower for the LB compared to wild type mice, indicating that abnormal mineralisation may therefore be associated with the impaired mechanical properties of muscles [28] in rachitic mice. This suggestion is supported by the increasing randomness of mineral particle orientation in the IF regions, which experience lower muscle forces, in both wild type and Hpr mice (Fig. 4A). This clear difference in mineralised nanostructure between the IF and LB may indicate the importance of the dynamic biomechanical stress environment for mineral particle rearrangement.

Furthermore, our results show striking differences in degree of orientation of mineral particles between the LB and IF regions (Fig. 4A), suggesting that spatial variances in mechanical environments within the same scapula surface may affect the degree of randomness of the mineralizing collagen fibre scaffold. In this regard, two systematic relationships were found in wild type animals. First, the increase of degree of orientation with developmental age is only seen in the LB. It has been shown previously that transfers of major muscle and joint forces take place predominantly through the thick bony ridges at the LB (22 MPa), but a lower force (7.5 MPa) is exerted on flat bony regions [5]. This strongly suggests that muscle mediated stress distributions associated with the orientation of the mineral phase at the nanometre length scale in flat bones. Furthermore, in 1 week old mice, there is no consistent increase in the degree of orientation from flat bony regions to bony ridges in scapula, which may be due to the low level of muscular force exerted on the bone in very young mice. Lastly, we suggest that the initial (1–4 weeks of development) rapid rate of increase in muscle weight, strength and muscle movement [28] in mice is associated with the initial rapid rate of increase of mineral particle alignment at the LB (Fig. 4A), and its subsequent stabilisation.

It is interesting, however, that this close relationship between muscle force and alignment in the wild type mice is far less prominent in Hpr mice. While the mineral particle degree of orientation does increase with age in Hpr animals, the clear differences in mineral crystal arrangement between bony ridges and flat bone regions are completely absent in the rickets. We propose that altered *in vivo* biomechanical forces are a deciding factor for these nanostructural differences. Extensive clinical evidence exists of altered muscular forces in rickets. Patients with X-linked hypophosphatemic rickets, roughly homologous to Hpr, have been reported to complain of muscle weakness, and X-linked hypophosphatemia has long term adverse effects on daily activities [26,29]. Furthermore, a study on another mouse model (Hyp) of X-linked hypophosphatemic rickets showed that grip strength and spontaneous movements of muscles were both affected in the diseased mice as opposed to wild type [28]. At the genetic level, the Hyp mice and Hpr mice both have *Phex* abnormalities [15]; it seems highly likely that Hpr mice will have similar musculoskeletal abnormalities to those reported for Hyp mice. Due to this skeletal muscle impairment in rachitic mice, *in vivo* forces on rachitic bones would be lower compared to wild-type tissue. Thus, altered muscular forces, arising due to the deficiency in phosphate levels in the serum, may be associated with nanostructural abnormalities in intramembranously ossifying bone.

These qualitative differences in mineral nanostructure are accompanied by quantitative differences in mineral concentration (measured by micro-CT) across the scapula bone, and deviation from this pattern in cases of metabolic disease. In wild type mice, the greater rate of increase of mineral concentration at the LB compared to the IF (Fig. 5A) indicates that a rapid increment in the mineral phase occurs at early stages of growth. This could be associated with faster muscle growth and elevated activity levels between 1 and 10 weeks developmental age in mice. At the flat bony IF, which experiences low force levels [5], the above observation is less pronounced (Fig. 5B). It has been demonstrated in other several studies that muscle strength has effects on bone mass or BMD that are independent of age, weight, height or drug usage [30–32]. Therefore, it is highly likely that muscle-mediated stress distributions influence spatial gradients in the nanostructure of the mineral phase, on a micrometre length scale. However, the clear difference in mineralisation between the IF and LB observed in wild type mice is quite absent in Hpr mice (Fig. 5A–B). The defective mineralisation in rachitic bone leads to a long-term reduced mineral content in full grown (10 week old) Hpr mice.

**Fig. 6:**
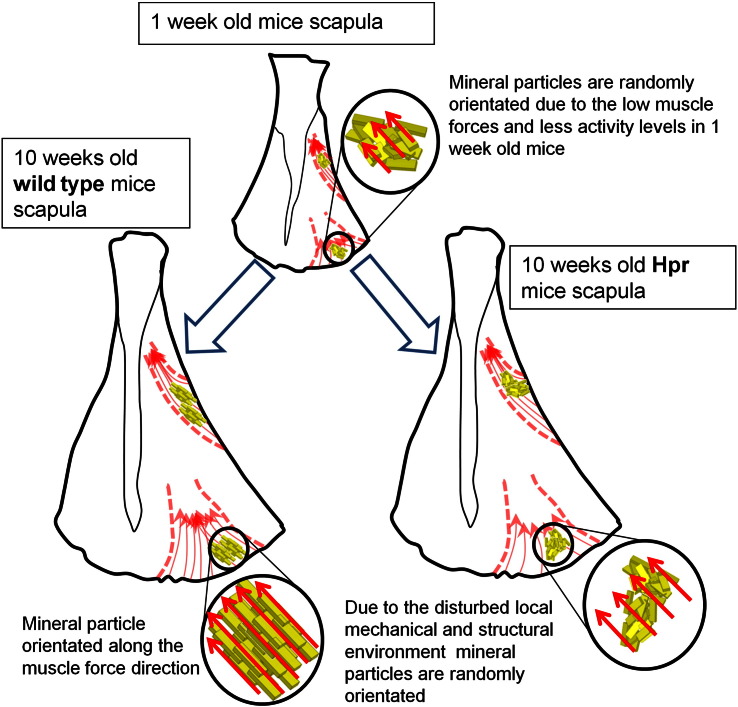
Model schematic showing development of macro structure, skeletal muscles and nanostructure of wild type and Hpr scapulae from 1 week to 10 weeks. As our experimental results (Figs. 2–5) showed, at 1 week the scapulae of both wild type and Hpr mice have similar nanostructural parameters, but this diverges considerably with increasing maturity due to insufficient muscle force loading on the Hpr bone.

**Fig. 7:**
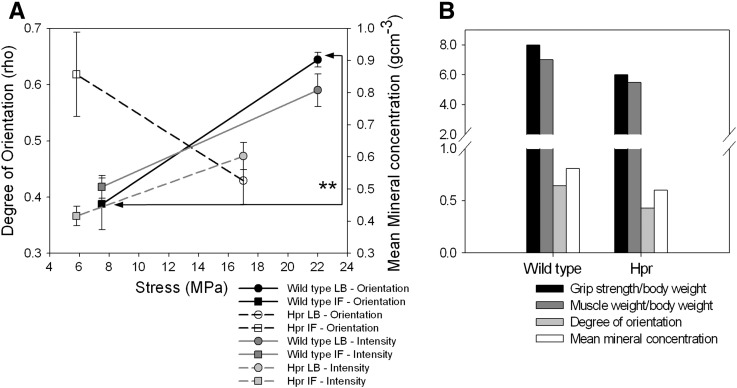
(A) Mineral particle degree of orientation (wild type: black symbols; Hpr: white symbols) and mean mineral content (wild-type: dark grey symbols; Hpr: light grey symbols) plotted against the localised stresses of the 10 week old mice scapulae. Stresses applied on the LB (circles) and IF (squares) of mature scapulae by shoulder muscles estimated by finite element modelling (data obtained from Gupta et al. 2004 [5]). Muscle strength was approximately 22.5% lower than those of the wild type mice in Hyp mice [28]. Therefore we assume, in our work, that stresses at LB and IF in Hpr mice scapula have been reduced by 22.5% in Hpr (Hpr and Hyp mice have similar abnormalities). (B) Comparison of bone mineral particle degree of orientation (light grey) and mean mineral concentration (white) with bicep muscle grip strength (black) and bicep muscle weight/body weight (dark grey) (obtained from Aono et al., 2011) for wild type and hyp mice. Pair-wise brackets denote statistical significance (*p < 0.05, **p < 0.01, ***p < 0.001, ns: not significant).

We propose a simple nano/microstructural model (Fig. 6) to correlate both the nanostructural mineral alignment and microstructural degree of mineralisation to altered muscular force distributions in rachitic bone. It is possible that the action of muscular stresses is linked to force-induced alignment of collagen fibrils and mineral crystals across the scapula bone, as hypothesised previously for long bones [2], followed by subsequent mineralisation. As bone mineral crystals have been reported to be deposited and to grow within the gap regions of the collagen type I fibrils under the influence of noncollageneous molecules [33], their orientation will follow the altered collagen fibrillar distribution. Previous calculations [5], via three-dimensional finite element modelling, showed a threefold higher stress level at the LB (22 MPa) compared to the IF (7.5 MPa) for healthy scapula bone. While the stress distribution on rachitic scapulae has not been studied computationally or experimentally, the skeletal muscle histology and physical performance in rickets have been well investigated [26,28,29,34]. These reports suggest progressive weakness and wasting of skeletal muscle in clinical and murine model studies of X-linked hypophosphatemic rickets; Hyp mice quadriceps muscle weight/body weight and strength/body weight have been measured to be approximately 20–25% smaller than wild type mice [28]. Therefore we hypothesise, in our model, that stresses at the LB and IF in the scapula have been similarly reduced by an average 22.5% in our Hpr mice (Fig. 7(A) open symbols).

Using this information on altered muscle strength and weight, we can correlate the stress distribution in the scapula, and its alterations during rickets, to the evolving mineral particle nanostructure. We plotted mineral particle alignment and degree of mineralisation versus applied stress for the oldest (10 weeks) wild type and Hpr mice in our data set. In wild type mice we observed a statistically significant (*p* < 0.05; Student's *t*-test) difference in mineral degree of orientation between two sites where low and high stresses are expected (Fig. 7(A)). Regions with expected increased stresses on the scapula are associated with an increased degree of mineral particle orientation, as well as mean mineral concentration. However, in Hpr mice, alterations in nanostructural properties are affected by the combined result of weaker skeletal muscles and impaired mineralisation. We used the reported grip strength (per unit body weight) of murine model called Hyp homologues of X-linked hypophosphatemic rickets at the same age (10 weeks) [28] as a proxy for muscular force, and compared our experimental data (degree of orientation) with the normalised grip strength and muscle weight for wild type and Hyp mice (Fig. 7(B)). A positive association between the two muscular parameters (grip strength and muscle weight) and the two mineralisation parameters (degree of orientation and mean mineral concentration) was observed. The grip strength, muscle weight, mineral particle degree of orientation and mineral concentration of Hyp mice were all lower than those of wild type mice. This finding suggests that abnormal changes in muscle forces in disease conditions are associated with reductions in the degree of mineral particle alignment.

To demonstrate the link between increasing muscular force and the development of nano-structural parameters requires that greater muscle strength/mass is associated with higher degree of mineral particle orientation, independent of body size variations. In this study we have demonstrated that degree of orientation, predominant orientation and mean mineral content increases with age in intramembranously ossifying bone. Several previous studies have shown a positive correlation between muscle strength and bone strength index [35] and BMD [30,31,36] independent of measures of body size. However, the correlation between development of muscle strength and nano-structural parameters was not investigated. Even though stress distribution in the developing scapula has not been studied, forces exerted by the mice with development (1 week to 4 weeks) in forelimbs have been studied using a dynamometer [27]. This study showed that muscular forces increase with age. This development of muscular forces may be linked to our observed time course of the development nano-structural parameters of mineral particle orientation (Fig. 3 and 4) and degree of mineralisation (Fig. 5).

## 5 Conclusion

The association between muscle strength and bone mass has been established in numerous studies [37–39], and mechanical stimulation by skeletal muscles has been reported to have a dominant effect on bone gain and loss when compared to non-mechanical factors such as hormones and metabolic environments [40,41]. This is further illustrated by the increased fracture risk and deformability observed in patients with muscle wasting and neuromuscular diseases such as muscular dystrophy, which implies an underlying altered bone material structure [42]. Furthermore, it has been shown that increasing muscle strength through exercise can reduce the risk of fracture and the development of kyphosis in older women with osteoporosis [43]. It has been demonstrated that increased fracture risk in the case of ageing bone is associated with changes in bone material [44] as well as reduced bone mass. To better understand the mechanisms in the bone material that mediate the alterations in gross fracture risk and deformability in metabolic bone disease, we have investigated mice with X-linked hypophosphatemic rickets, a disease that is associated with progressive weakness and wasting of skeletal muscle [45] as well as a reduction in lowered bone mineral content. In this rachitic condition, deterioration in the skeletal muscle increases the deformability and fracture of bone. Our results show that alterations in the nanostructure of the bone matrix – such as the direction and degree of mineral particle orientation – are associated with both predicted reduction in muscle forces and altered mineralisation in the disease condition. Hence, we propose that the nanostructural parameters of mineral particle orientation and direction may play a vital role in controlling the fracture risk and the deformability in the bone tissue. Furthermore, the nanostructural parameters like the degree of orientation and mineral particle angle could potentially be used as markers to estimate the fracture risk and the deformability in bone in metabolic and neuromuscular bone diseases.
